# Treatment efficacy for infantile epileptic spasms syndrome in children with trisomy 21

**DOI:** 10.3389/fped.2025.1498425

**Published:** 2025-02-12

**Authors:** Henry Chen, Adam L. Numis, Renée A. Shellhaas, John R. Mytinger, Debopam Samanta, Rani K. Singh, Shaun A. Hussain, Danielle Takacs, Kelly G. Knupp, Li-Rong Shao, Carl E. Stafstrom

**Affiliations:** ^1^ Department of Neurology and Weill Institute for Neuroscience, University of California San Francisco, San Francisco, CA, United States; ^2^ Department of Pediatrics, UCSF Benioff Children’s Hospital, University of California San Francisco, San Francisco, CA, United States; ^3^ Department of Neurology, Washington University at St. Louis, St. Louis, MO, United States; ^4^ Division of Pediatric Neurology, Department of Pediatrics, Nationwide Children’s Hospital, The Ohio State University, Columbus, OH, United States; ^5^ Department of Pediatrics, University of Arkansas for Medical Sciences, Little Rock, AR, United States; ^6^ Department of Pediatrics, Atrium Health-Levine Children’s Hospital, Charlotte, NC, United States; ^7^ Department of Pediatrics, Wake Forest University School of Medicine, Winston-Salem, NC, United States; ^8^ Division of Pediatric Neurology, Department of Pediatrics, University of California, Los Angeles, CA, United States; ^9^ Department of Pediatric Neurology and Developmental Neuroscience, Texas Children’s Hospital, Baylor College of Medicine, Houston, TX, United States; ^10^ Departments of Pediatrics and Neurology, University of Colorado Anschutz Medical Campus, Aurora, CO, United States; ^11^ Division of Pediatric Neurology, Department of Neurology, Johns Hopkins University, Baltimore, MD, United States

**Keywords:** infantile spasms, infantile epileptic spasms syndrome, Down syndrome, trisomy 21, anti-seizure medications, hypsarrhythmia

## Abstract

**Background:**

Infantile Epileptic Spasms Syndrome (IESS) is the most common epilepsy syndrome in children with trisomy 21. First-line standard treatments for IESS include adrenocorticotropic hormone (ACTH), oral corticosteroids, and vigabatrin. Among children with trisomy 21 and IESS, treatment with ACTH or oral corticosteroids may yield higher response rates compared with vigabatrin. However, supporting data are largely from single-center, retrospective cohort studies.

**Methods:**

Leveraging the multi-center, prospective National Infantile Spasms Consortium (NISC) database, we evaluated the efficacy of first-line (standard) treatments for IESS in children with trisomy 21. We assessed clinical spasms remission at two weeks, clinical spasms remission at three months, and improvement of EEG (resolution of hypsarrhythmia) three months after initiation of treatment.

**Results:**

Thirty four of 644 (5.3%) children with IESS were diagnosed with trisomy 21. In all children with trisomy 21, epileptic spasms was their presenting seizure type. Twenty of 34 (59%) children were initially treated with ACTH, nine (26%) with oral corticosteroids, and five (15%) with vigabatrin. Baseline demographics did not vary among treatment groups. The overall clinical remission rate after two weeks of treatment was 53% including 13 of 20 (65%) receiving ACTH, three of nine (33%) receiving oral corticosteroids, and two of five (40%) receiving vigabatrin (*p* = 0.24). The continued clinical response rate at three months was 32% including 8 of 20 (40%) receiving ACTH, two of nine (22%) receiving oral corticosteroids, and one of five (20%) receiving vigabatrin. Thirty of the 34 (88%) children presented with hypsarrhythmia (88%). EEG improvement at three months was better for children treated with ACTH (74%) or oral corticosteroids (83%) than vigabatrin (20%; *p* = 0.048). Adjustment for time from epileptic spasms onset to treatment did not alter results.

**Conclusions:**

In our cohort, epileptic spasms were the first presenting seizure type in all children with trisomy 21. Among first-line standard treatment options, ACTH may have superior efficacy for clinical and electrographic outcomes for IESS in children with trisomy 21.

## Highlights

• Epileptic spasms were the first recognized seizure type in all children with trisomy 21.
• Children with trisomy 21 who were treated with ACTH first had higher responder rates at 2 weeks and 3 months compared with oral corticosteroids and vigabatrin.
• Hypsarrhythmia resolution rate at 3 months was higher in children who received ACTH or oral corticosteroids than those who received vigabatrin.

## Introduction

Trisomy 21 (Down syndrome) is the most prevalent genetic disorder caused by a chromosomal abnormality and 5%–10% of affected children develop epilepsy (1, 2). Infantile Epileptic Spasms Syndrome (IESS) is the most common epilepsy syndrome in children with trisomy 21 with a lifetime prevalence estimate of 0.4%–12.8% (3–5). IESS typically presents as ictal flexor or extensor epileptic spasms with an electroencephalogram (EEG) that often exhibits the interictal pattern of hypsarrhythmia (6, 7). IESS can be associated with developmental stagnation or regression regardless of etiology (8). While children with trisomy 21 and IESS have been reported to respond favorably to treatment compared with spasms in the non-trisomy 21 population (3, 4, 9), epileptic spasms may exacerbate developmental deficits in children with Down syndrome. Therefore, early, effective treatment is critical to optimize neurodevelopmental outcomes (10, 11) and it is imperative to identify the most effective treatment strategy for this high-risk population (12).

Standard, first-line medications for the treatment of IESS include adrenocorticotrophic hormone (ACTH), oral corticosteroids (prednisone or prednisolone), and vigabatrin (13–18). Treatment choice and response rates can vary by etiology of IESS [i.e., children with IESS caused by tuberous sclerosis complex respond best to vigabatrin; (19, 20)]. However, the optimal choice of first-line therapy for IESS in children with trisomy 21 remains uncertain. Prior retrospective, single-center studies have reported trends toward higher response rates when treatment is initiated with hormonal therapies (ACTH or oral corticosteroids) compared with vigabatrin and nonstandard therapies (5, 21).

Here, we evaluate the efficacy of standard, first-line medications for the treatment of IESS in children with trisomy 21 using data from a multi-site, prospective database, the National Infantile Spasms Consortium (NISC). Using NISC, we examined early and sustained treatment responses by evaluating clinical remission of epileptic spasms (at 2 weeks and 3 months) and electrographic remission of hypsarrhythmia (at 3 months) (22). We hypothesized that treatment response trends in the trisomy 21 population would resemble the overall NISC population, with hormonal therapy being most efficacious for first-line treatment for children with IESS associated with trisomy 21.

## Methods

We used a nested cohort design within the NISC dataset. NISC is a national 22-center prospective study developed by the Pediatric Epilepsy Research Consortium (PERC) to evaluate the efficacy of treatments in children with IESS from January 2012 to December 2018. Children presenting between the ages of two months and two years with new onset epileptic spasms were eligible for enrollment. Medication dosing recommendations for ACTH, oral corticosteroids, and vigabatrin were provided to all sites, but treatment decisions were made by the clinical team at each site. Detailed methods regarding data collection have been previously reported (22).

This study was approved by the Institutional Review Boards at all participating sites. Written informed consent was obtained from a parent or guardian of each enrolled child in accordance with site-specific institutional requirements. Data were collected through chart review entered in REDCap (REDCap Consortium; Nashville TN) (17, 22).

In this secondary analysis, we restricted the NISC cohort to children with a genetically confirmed diagnosis of trisomy 21. Baseline demographics were collected and the efficacy of first-line treatments for IESS was evaluated using clinical information collected 2 weeks and three months after treatment initiation. Given the low response rate to non-standard medications compared to standard medications (ACTH, corticosteroids, or vigabatrin) in the management of IESS, children who were initially treated with a non-standard first treatment were analyzed according to the first *standard* medication prescribed. Primary outcomes of IESS remission were measured at two time intervals: remission of clinical epileptic spasms at two weeks after initial standard treatment of IESS and continued remission of clinical epileptic spasms at three months after the initial standard treatment of IESS. Non-responders at three months included children with a lack of complete remission of spasms or relapse of clinical epileptic spasms.

We then evaluated the impact of treatment on hypsarrhythmia at three months (secondary outcome). In the NISC, hypsarrhythmia was defined as the presence of multifocal spikes, background disorganization, and background voltage >200 µV peak-to-peak in any epoch on a bipolar longitudinal montage, and was determined by the referring neurologist at each site (17). There was no consensus definition provided for modified hypsarrhythmia variants. With consideration of the low inter-rater reliability for hypsarrhythmia and modified hypsarrhythmia variants, we merged these two variables during analysis (23). An electroclinical response was defined as both resolved hypsarrhythmia on EEG and continued remission of spasms at three months.

To account for the potential impact of dosing variability on treatment response, we performed a sensitivity analysis on children receiving adequate doses of ACTH, oral corticosteroids, and vigabatrin. Within each treatment group, adequate dosing was defined as ACTH >140 U/m^2^, oral corticosteroids ≥40 mg/day, or vigabatrin ≥100 mg/kg/day (13, 24).

### Statistical analyses

Between-group comparisons of continuous variables were accomplished with Student's *t*-tests, analysis of variance (ANOVA), or Kruskal–Wallis tests, as appropriate. Comparisons of categorical variables were carried out with chi-square tests. We evaluated the effect of lag-time from diagnosis of IESS to treatment initiation on 2-week and 3-month responder rates using a generalized linear model with log-link regression and robust standard errors. All analyses were conducted using Stata version® 17 (College Station, Texas, USA). Statistical significance was defined as *p*-value less than 0.05. Given the small sample size of this cohort, we highlight large effect sizes that are not statistically significant in our results.

## Results

In the NISC cohort, 34 of 644 (5.3%) children with IESS were diagnosed with trisomy 21, and this was the most common genetic diagnosis in the IESS cohort. Thirteen NISC sites provided at least one child included in the trisomy 21 cohort. Epileptic spasms were the presenting seizure type in all children with trisomy 21 (e.g., none had pre-existing epilepsy when they presented with their first epileptic spasm). There was no difference in the age of spasms onset among the children with trisomy 21 [6.88 months; interquartile range (IQR): 6.0, 7.8] vs. the overall NISC cohort (6.91 months; IQR: 6.8, 7.2) (*p* = 0.51).

Among the 34 children with trisomy 21 and IESS, twenty (59%) were initially treated with ACTH, nine (26%) with oral corticosteroids, and five (15%) with vigabatrin. One child had previously received zonisamide for 108 days for IESS and then due to inefficacy, was switched to ACTH. No other child received a non-standard medication as initial treatment of IESS.

We did not observe differences in demographics across treatment groups (Table 1). Due to limitations of our sample size, we were unable to adjust for demographic confounders. Thirty of 34 children (88%) had hypsarrhythmia on EEG and 14 children (56%) were diagnosed with developmental regression at the time of presentation. The median time from epileptic spasms onset to the administration of a standard IESS treatment was 27 days (IQR: 9, 64). Median lag-time from diagnosis of IESS to treatment initiation was longer in participants initially treated with ACTH (32 days) compared with patients treated with vigabatrin (16 days) or oral corticosteroids (21 days), though differences did not reach statistical significance (*p* = 0.47, Table 1).

**Table 1:** Baseline characteristics of 34 children with trisomy 21 and infantile epileptic spasms syndrome.

Infant Characteristics	Total *N* = 34	Initial Treatment for IESS			*p*-value
		ACTH *N* = 20	Corticosteroids *N* = 9	Vigabatrin *N* = 5	
Sex, *n* (%)					
Male	22 (65)	13 (65)	6 (67)	3 (60)	0.49
Race, *n* (%)					
Asian	2 (6)	1 (5)	1 (11)	0 (0)	0.29
Black	4 (12)	2 (10)	0	2 (40)	
White	23 (68)	15 (75)	6 (67)	2 (40)	
Other or unknown	5 (15)	2 (10)	2 (22)	1 (20)	
Ethnicity, *n* (%)					
Hispanic	4 (12)	1 (5)	2 (22)	1 (20)	0.18
Insurance Class, *n* (%)					
Public	10 (29)	5 (25)	2 (22)	3 (60)	0.77
Private	19 (56)	13 (65)	5 (56)	1 (20)	
Other or unknown	5 (15)	2 (10)	2 (22)	1 (20)	
Distance to Epilepsy Center, *n* (%)					
Same city	4 (12)	3 (15)	1 (11)	0 (0)	0.53
Outside of city (<100 miles away)	23 (68)	12 (60)	8 (89)	3 (60)	
Outside of city (>100 miles away)	7 (21)	5 (25)	0 (0)	2 (40)	
History of seizures prior to IESS diagnosis, *n* (%)	0 (0)	0 (0)	0 (0)	0 (0)	1.0
Gestational Age at birth (weeks), median (IQR)	37 (36, 39)	37 (36, 39)	37 (36, 40)	40.0 (39, 40)	0.07
Age at epileptic spasms onset (months), median (IQR)	6.0 (5.5, 8.2)	6.0 (5.1, 7.5)	7.0 (6.5, 11.5)	6.0 (5.5, 6.0)	0.14
Lag Time to treatment initiation (days), median (IQR)	27 (9, 64)	32 (12, 84)	21 (5, 61)	16 (4, 35)	0.47
Baseline Hypsarrhythmia, *n* (%)					
Yes, including Modified Variants	30 (88)	19	6	5	
Baseline Developmental Regression, *n* (%)					
Definite or Possible	14 (56)	6 (43)	7 (88)	1 (33)	0.60
No	11 (44)	8 (57)	1 (13)	2 (67)	

Abbreviations: ACTH, adrenocorticotropic hormone; IESS, infantile epileptic spasms syndrome.

On the participants for which brain magnetic resonance imaging (MRI) scans were obtained, findings were either normal or showed mild cortical, cerebellar, or brainstem hypoplasia, as previously shown for children with trisomy 21 (25). Vigabatrin-related gray matter diffusion restriction was not seen (26).

Side effects of ACTH, oral corticosteroids, and vigabatrin observed in our trisomy 21 patients were similar to those of the overall NISC cohort. Among those treated with ACTH or oral corticosteroids, weight gain, hypertension, and mild irritability were the most common adverse effects, and none were sufficiently severe to warrant medication discontinuation. Likewise, some children treated with vigabatrin showed mild irritability or sedation; there were no concerns about vision impairment during the brief follow up period.

**Figure 1:**
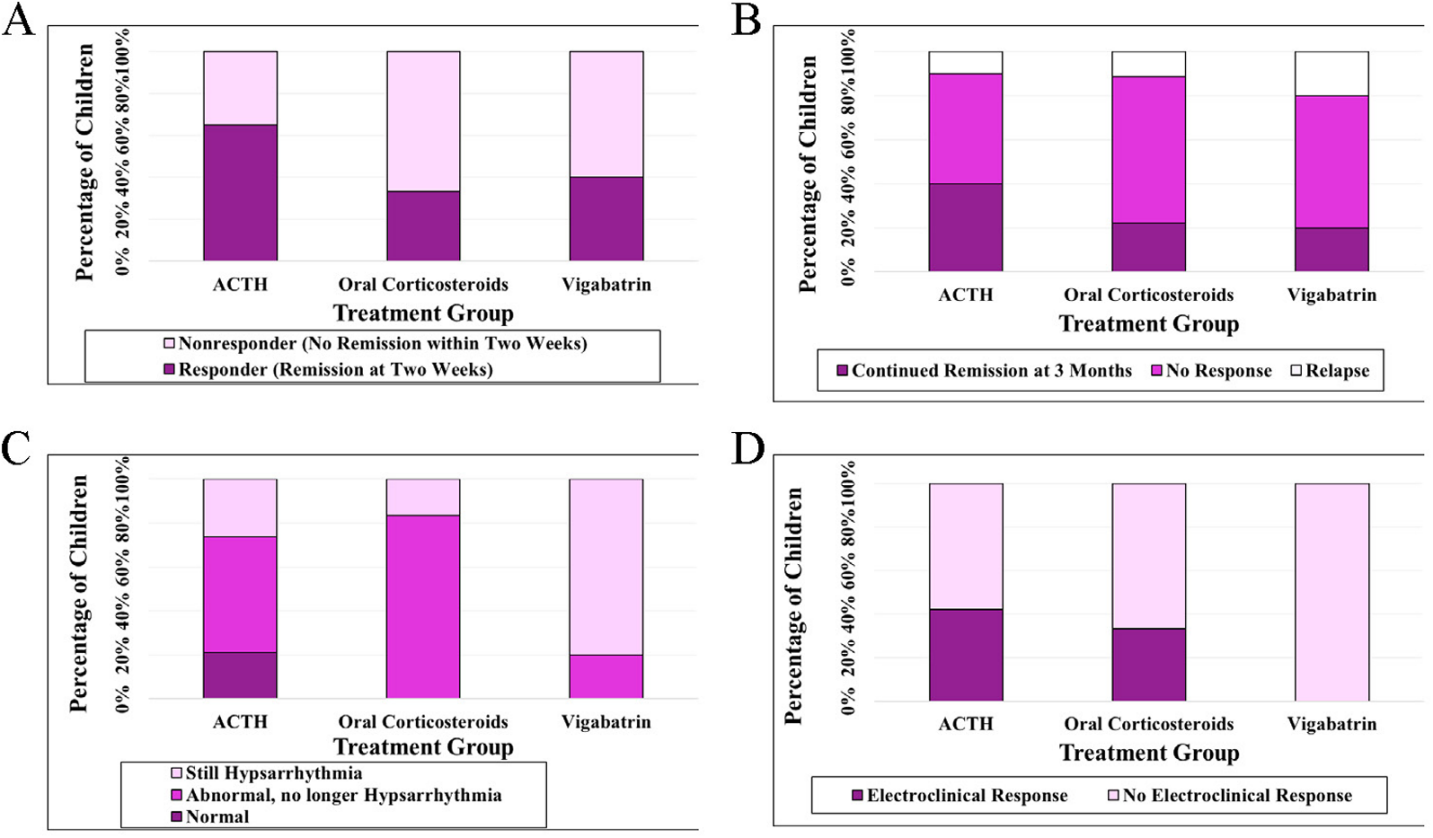
Treatment response at 2 weeks **(A)** and 3 months **(B)** for infantile epileptic spasms syndrome in children with trisomy 21, association with EEG improvement **(C)**, and association with combined EEG improvement and clinical response at 3 months **(D)**.

Two weeks after initiation of a standard therapy, epileptic spasms resolved in 18 of 34 (53%) children. Responders at two weeks included 13 of 20 (65%) receiving ACTH, three of nine (33%) receiving oral corticosteroids, and two of five (40%) receiving vigabatrin (*p* = 0.24; Figure 1A, Table 2A). In children who responded to treatment at two weeks, relapse occurred in two of thirteen (15%) children who initially responded to ACTH, one of three children (33%) who initially responded to corticosteroids, and one of two children (50%) who initially responded to vigabatrin.

**Table 2A:** Clinical responses at 2 weeks and 3 months.

Treatment	Responders at 2 weeks	Relapsers between 2 weeks and 3 months	Responders at 3 months including relapsers
ACTH	13/20 (65%)	2/13 (15%)	8/20 (40%)
OCS	3/9 (33%)	1/3 (33%)	2/9 (22%)
VGB	2/5 (40%)	1/2 (50%)	1/5 (20%)

Of children with clinical remission at 2 weeks, there was continued remission of epileptic spasms in 9 of 18 (50%) children at 3 months, including seven of 13 (54%) receiving ACTH, one of three receiving oral corticosteroids (33%), and one of two (50%) receiving vigabatrin (not significant) (Table 2A). Irrespective of 2-week remission, clinical remission of epileptic spasms at three months was reported in 11 of 34 children (32%). These clinical responders at three months included 8 of 20 (40%) receiving ACTH, two of nine (22%) receiving oral corticosteroids, and one of five (20%) receiving vigabatrin (*p* = 0.52; Figure 1B; Table 2A). A similar non-significant result was seen if the ACTH cohort and the corticosteroid cohort were combined and compared with vigabatrin (*p* = 0.47).

Among the 30 children that presented with hypsarrhythmia at the time of IESS diagnosis, EEG improvement at three months was seen in 14 of 19 (74%) children receiving ACTH, five of six (83%) receiving oral corticosteroids, and one of five (20%) receiving vigabatrin (*p* = 0.03 comparing ACTH and oral corticosteroids with vigabatrin; Figure 1C; Table 2B). The EEG of four children (13%) with hypsarrhythmia at diagnosis of IESS normalized at three months; all of these children were treated with ACTH.

**Table 2B:** EEG improvement at 3 months in subjects presenting with hypsarrhythmia at time of diagnosis: *n* = 30.

	Overall improvement (Normal or Abnormal but not Hypsarrhythmia)	Hypsarrhythmia	Improvement both clinical and EEG
ACTH	14/19 (74%)	5/19 (26%)	8/19 (42%)
OCS	5/6 (83%)	1/6 (17%)	2/6 (33%)
VGB	1/5 (20%)	5/6 (83%)	0/5 (0%)

OCS, oral corticosteroids; VGB, vigabatrin.

When accounting for both EEG improvement and clinical remission at 3 months, an electroclinical response was observed in 10 of the 30 (33%) children who initially presented with hypsarrhythmia. Clinical remission without EEG improvement was observed in one child receiving vigabatrin. Electroclinical response was observed in a similar percentage of children receiving ACTH or oral corticosteroids: eight of 19 (42%) receiving ACTH and two of six (33%) receiving oral corticosteroids. Electroclinical response was not observed in any of the five children receiving vigabatrin (*p* = 0.14 comparing ACTH and oral corticosteroids to vigabatrin; Figure 1D). Adjustment for lag-time from diagnosis of IESS to treatment initiation by treatment group did not alter two-week or three-month results.

**Figure 2:**
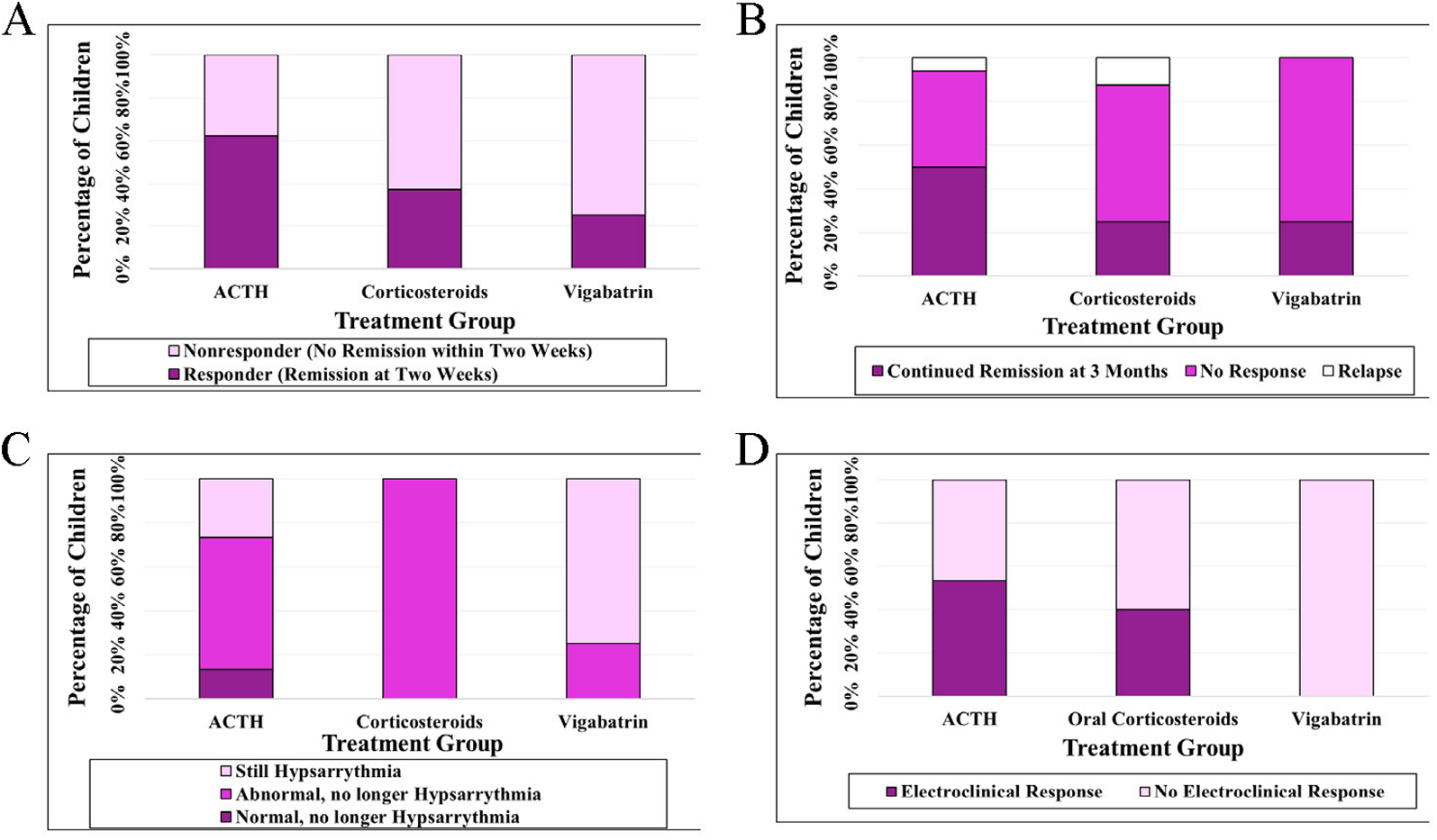
Treatment response at 2 weeks **(A)** and 3 months **(B)** for infantile epileptic spasms syndrome in children with trisomy 21 receiving adequate doses of first-line therapies, and association with EEG improvement **(C)**, and association with combined EEG improvement and clinical response at 3 months **(D)**.

In a sensitivity analysis, we restricted our analysis to children who received adequate treatment doses. Adequate doses of initial standard treatments were given in 16 of 20 (80%) children receiving ACTH, eight of nine (89%) children receiving corticosteroids, and four of five (80%) children receiving vigabatrin. Results from this sensitivity analysis did not alter our other findings (Figure 2).

## Discussion

In this multi-center prospective cohort study, we evaluated the outcomes of IESS treatments in children with trisomy 21. Our findings suggest that treatment with hormonal therapy (ACTH or oral corticosteroids) compared to vigabatrin result in an improved response at two weeks and three months after treatment initiation and improved resolution of hypsarrhythmia (at three months). The absence of electroclinical responders among children receiving vigabatrin may suggest that hormonal therapies are more effective than vigabatrin at improving clinical and electrographic outcomes, although the small sample size precludes precise estimates.

The 2-week and 3-month responder rates (53% and 32%) for children with trisomy 21 were similar to the overall NISC cohort responder rate (46%), and included both early and late responders. Likewise, the highest responder rates at two weeks were observed in children treated with ACTH, followed by oral corticosteroids and vigabatrin (22). Prior reports found ACTH and oral corticosteroids to have roughly similar effectiveness in IESS overall (17) and the lower response rates to oral steroids here was likely due to underpowering. Our findings on clinical and electrographic response to standard therapies corroborate the findings of several other groups that hormonal therapies are superior to vigabatrin for IESS among children with trisomy 21 (5, 21, 27–29).

Further support for that conclusion comes from a recent retrospective cohort study from Ireland of 54 children with IESS and trisomy 21, in which ACTH was underrepresented (only one of the 54 children was treated with ACTH) (26). A higher response rate was found among children treated with corticosteroids (60% had spasms cessation) relative to those treated with vigabatrin (28% had spasms cessation), though treatment with a combination of prednisolone and vigabatrin afforded an 83% spasms cessation rate. Despite some methodological differences between these studies, they reveal trends towards higher response rates to oral corticosteroids relative to vigabatrin, supporting the enlarging literature that hormonal therapy may be a more effective first-line therapy than vigabatrin for children with IESS in the setting of trisomy 21. Of note, a large retrospective review of treatments of infantile spasms across etiologies found a beneficial effect of vigabatrin or vigabatrin plus steroids (but only two of the 198 subjects had trisomy 21) (18). In the larger International Collaborative Infantile Spasms Study (ICISS) that included 37 children with infantile spasms and trisomy 21, those treated with prednisolone responded similarly to those treated with prednisolone plus vigabatrin, suggesting the lack of an additional benefit of vigabatrin (30).

Previous studies reported that diagnosis and treatment of IESS in children with trisomy 21 is often delayed compared to other children diagnosed with IESS (31). While the treatment lag-time (measured as the time from epileptic spasms onset to treatment initiation) in our cohort was shorter than that observed in previous studies in children with trisomy 21 and IESS, there remains a greater treatment lag-time relative to the general cohort in NISC [27 days (IQR: 9, 64) vs. 15 days (IQR: 6, 37)] (21, 31). Other studies have shown that shorter treatment lag-time may lead to improved IESS treatment efficacy (32, 33). Interestingly, in our study, children treated with ACTH had the longest lag times, yet maintained the highest responder rate across all standard therapies. Prompt diagnosis and efficient treatment initiation could provide opportunities for better outcomes (16).

Importantly, we observed that epileptic spasms were the presenting seizure type for all children with trisomy 21. That is, none of the children presented with focal seizures or other seizure types prior to infantile spasms, an observation that may be relevant to the mechanisms underlying predisposition to infantile spasms in children with trisomy 21 (34). In consideration of the challenges to prompt diagnosis and medical intervention for IESS in this population, our findings underscore the need for early epilepsy counseling of families of children with trisomy 21, at birth or soon thereafter (31, 35). IESS is a “never miss” diagnosis for pediatricians; our results emphasize the need for education of primary care providers about semiology of epileptic spasms and the urgency of diagnosis and treatment. Proactive counseling of families with infants at high risk for IESS (including children with trisomy 21) could include recommendations to record any suspicious events with a smartphone video and vigilant attention to any plateau or regression of developmental milestones (36, 37).

Historically, the low prevalence of children with both IESS and trisomy 21 has posed challenges to designing studies with adequate sample sizes and statistical power (5). In leveraging the multi-center prospective NISC cohort, we reduce the potential effect of site-specific treatment and selection bias inherent in single-center retrospective studies. Yet, despite enrolling from sites across the United States, NISC was still limited by a small sample size in this relatively rare disease combination. Collaboration between international consortia may be necessary to carry out multi-center prospective studies with larger sample sizes. Alternatively, use of a learning healthcare system model of research and quality improvement may offer an informative approach to case identification and research optimization for these children (38). Such efforts would provide further confidence in our results and strengthen understanding of the natural history of IESS in children with trisomy 21 and the relative efficacy of treatment options for this population.

We were also limited by our methodology for assessing electrographic changes on EEG. Neurologists at each NISC site classified EEGs as hypsarrhythmia or a modified variant of hypsarrhythmia. Since these data were collected, hypsarrhythmia assessments have been shown to demonstrate poor inter-rater reliability (39). To mitigate risks of EEG measurement validity, future studies could consider adopting the Burden of AmplitudeS and Epileptic Discharges (BASED) score, an EEG scoring system with demonstrated inter-rater validity or a centralized EEG review process to improve EEG assessment validity and accuracy (40, 41).

At this time, the optimal treatment choice for trisomy 21 children with IESS remains unclear. Our data support hormonal treatments but ACTH and corticosteroids are nonspecific with regard to mechanism and are also used as the first treatment choice for IESS of most other etiologies. MRI scan findings and potential side effects are not unique to the trisomy 21 population and MRI results did not alter the treatment approach in prior studies (25). Therefore, the choice of treatment can be informed by efficacy considerations.

## Conclusion

In our cohort, epileptic spasms were the first presenting seizure type in all children with trisomy 21. Among first-line standard treatment options, hormonal therapies (and perhaps ACTH in particular) appear to have better efficacy than vigabatrin for clinical and electrographic outcomes of IESS in children with trisomy 21.

## Data Availability

The raw data supporting the conclusions of this article will be made available by the authors, without undue reservation.
